# Impaired HMG-CoA Reductase Activity Caused by Genetic Variants or Statin Exposure: Impact on Human Adipose Tissue, β-Cells and Metabolome

**DOI:** 10.3390/metabo11090574

**Published:** 2021-08-25

**Authors:** Assel Sarsenbayeva, Bipasha Nandi Jui, Giovanni Fanni, Pedro Barbosa, Fozia Ahmed, Robin Kristófi, Jing Cen, Azazul Chowdhury, Stanko Skrtic, Peter Bergsten, Tove Fall, Jan W. Eriksson, Maria J. Pereira

**Affiliations:** 1 Department of Medical Sciences, Clinical Diabetology and Metabolism, Uppsala University, 751 85 Uppsala, Sweden; assel.sarsenbayeva@medsci.uu.se (A.S.); Bipasha.Nandijui.2754@student.uu.se (B.N.J.); giovanni.fanni@medsci.uu.se (G.F.); fozia.ahmed@medsci.uu.se (F.A.); robin.kristofi@medsci.uu.se (R.K.); tove.fall@medsci.uu.se (T.F.); jan.eriksson@medsci.uu.se (J.W.E.); 2 Center for Neuroscience and Cell Biology, University of Coimbra, 3004-504 Coimbra, Portugal; pedrobarbosa13@hotmail.com; 3 Institute for Interdisciplinary Research, University of Coimbra, 3030-789 Coimbra, Portugal; 4 Department of Medical Cell Biology, Uppsala University, 751 85 Uppsala, Sweden; jing.cen@mcb.uu.se (J.C.); azazul.chowdhury@mcb.uu.se (A.C.); peter.bergsten@mcb.uu.se (P.B.); 5 Innovation Strategies & External Liaison, Pharmaceutical Technologies & Development, AstraZeneca, 431 83 Gothenburg, Sweden; Stanko.Skrtic@astrazeneca.com; 6 Institute of Medicine at Sahlgrenska Academy, University of Gothenburg, 413 45 Gothenburg, Sweden

**Keywords:** statins, adipose tissue, β-cell, insulin resistance, glucose uptake, type 2 diabetes

## Abstract

Inhibition of 3-hydroxy-3-methyl-glutaryl-CoA (HMG-CoA) reductase is associated with an increased risk of new-onset type 2 diabetes. We studied the association of genetic or pharmacological HMG-CoA reductase inhibition with plasma and adipose tissue (AT) metabolome and AT metabolic pathways. We also investigated the effects of statin-mediated pharmacological inhibition of HMG-CoA reductase on systemic insulin sensitivity by measuring the HOMA-IR index in subjects with or without statin therapy. The direct effects of simvastatin (20–250 nM) or its active metabolite simvastatin hydroxy acid (SA) (8–30 nM) were investigated on human adipocyte glucose uptake, lipolysis, and differentiation and pancreatic insulin secretion. We observed that the LDL-lowering *HMGCR* rs12916-T allele was negatively associated with plasma phosphatidylcholines and sphingomyelins, and *HMGCR* expression in AT was correlated with various metabolic and mitochondrial pathways. Clinical data showed that statin treatment was associated with HOMA-IR index after adjustment for age, sex, BMI, HbA1c, LDL-c levels, and diabetes status in the subjects. Supra-therapeutic concentrations of simvastatin reduced glucose uptake in adipocytes and normalized fatty acid-induced insulin hypersecretion from β-cells. Our data suggest that inhibition of HMG-CoA reductase is associated with insulin resistance. However, statins have a very mild direct effect on AT and pancreas, hence, other tissues as the liver or muscle appear to be of greater importance.

## 1. Introduction

3-hydroxy-3-methyl-glutaryl-CoA reductase (HMG-CoA reductase, *HMGCR*) is a rate-limiting enzyme involved in the mevalonate pathway responsible for the synthesis of cholesterol, which is a vital precursor of different biologically active molecules, such as steroid hormones and lipoproteins [1]. Statins are lipid-lowering drugs, which potently inhibit the activity of HMG-CoA reductase and effectively reduce the plasma concentration of LDL cholesterol. Since statins reduce the risk of cardiovascular events and overall mortality in the patients [2], they are one of the most widely prescribed drugs in the world. For instance, in the USA, the number of adults taking statins increased from 21.8 million in 2002–2003 to 39.2 million in 2012–2013 [3]. Apart from reducing plasma cholesterol levels, statins inhibit the synthesis of other components of the mevalonate pathway, such as major isoprenoids, farnesyl and geranylgeranyl pyrophosphate [1,4]. Aberrant synthesis of these compounds contributes to the development of different disorders, including cancer, cerebrovascular diseases, and Alzheimer’s disease [4]. Statins’ anti-neoplastic, anti-inflammatory, and cardioprotective functions make them a potential therapeutic agent for these conditions [5]. Despite being generally safe and well-tolerated, statin therapy is associated with an increased risk of new-onset type 2 diabetes (T2D) [6]. Several pre-existing risk factors can stimulate the diabetogenic potential of the therapy, including therapy intensity, some genetic predisposition, sex, and age of the patients [6,7,8,9,10]. Statins with high or moderate potency, such as atorvastatin and simvastatin, are reported to have a higher propensity to induce new-onset diabetes in patients compared to low potency statins, e.g., fluvastatin [9]. Comparison between statins indicated that patients taking atorvastatin or simvastatin have increased risk of new-onset diabetes [9], reduced insulin secretion, and insulin sensitivity [11]. On the other hand, pravastatin is considered protective against diabetes development [9,12,13]. This could be due to the lipophilicity of the drugs, as lipophilic statins (e.g., atorvastatin and simvastatin) can pass through the cell membrane of extrahepatic tissues, such as the pancreas, unlike hydrophilic statins (pravastatin) [14], which could contribute to their diabetogenic effect.

Additionally, the rs12916 *HMGCR* polymorphism is associated with reduced hepatic *HMGCR* expression and reduced LDL-cholesterol levels, as well as increased diabetes risk [15]. Genetic inhibition of HMG-CoA reductase due to this polymorphism appears to resemble metabolic side effects of statin therapy. Therefore, it is of interest to understand whether the association of *HMGCR* and its LDL-lowering polymorphism with certain plasma or adipose tissue metabolites could give a closer insight into the diabetogenic effects of statins. 

Several possible mechanisms for the diabetogenic actions of statins have been proposed. Pancreatic β-cells are capable of de novo cholesterol synthesis. Inhibition of cholesterol production leads to deprivation of plasma membrane cholesterol, increased LDL receptor expression and increased uptake of plasma LDL in the β-cells [16,17]. Accumulating LDL in the β-cells from the bloodstream seems to induce inflammation and apoptosis of mice and human pancreatic β-cells and, hence, impair their function [16,18]. In vitro and animal studies on skeletal muscle cells have shown that simvastatin reduced glucose transporter 4 (GLUT4) translocation and impaired glucose uptake via interfering with insulin signaling cascade [19,20]. Animal studies have also demonstrated that the mevalonate pathway and its components seem to be highly important for the metabolic functions of adipose tissue [21,22]. Studies have also demonstrated that supra-therapeutic concentrations of statins (atorvastatin and simvastatin) inhibited 3T3-L1 adipocyte differentiation and GLUT4 translocation in adipocytes as well [23,24]. Interestingly, most studies have been performed in vitro using concentrations of statins in the range of 1–50 µM, which is more than 1000 times higher than the concentrations seen in human plasma [25]. Similarly, the authors report that animal studies also use a much higher dose than the dose administered to the patients (1–100 mg/kg vs. 0.1–1 mg/kg, respectively) [25]. Therefore, studies with relevant therapeutic concentrations are warranted. Finally, most of the studies use simvastatin lactone, which is an inactive prodrug [25], therefore, its active metabolite, simvastatin hydroxy acid, also needs to be tested.

We hypothesize that inhibition of HMG-CoA reductase has a negative impact on plasma and adipose tissue metabolome and human adipose tissue metabolism, and pancreatic islet function. Therefore, our work aims to study the direct effects of pharmacological inhibition of HMG-CoA reductase with statins (simvastatin and its active metabolite simvastatin hydroxy acid) on human subcutaneous adipose tissue and isolated human pancreatic islet function. The concentration of the drugs was selected according to the reported therapeutic concentrations to perform clinically relevant experiments. Furthermore, we investigated the impact of a genetic *HMGCR* variant lowering LDL-cholesterol levels on the circulating metabolome. 

## 2. Results

We aimed to study the association of HMG-CoA reductase with adipose tissue and plasma metabolome and to understand whether genetic and pharmacological inhibition of the enzyme would affect the circulating metabolome or aggravate whole-body metabolism. For this, we performed the following human studies.

### 2.1. Association Studies

#### 2.1.1. Association of Expression of *HMGCR* and Other Genes and Metabolites in Adipose Tissue

**Figure 1:**
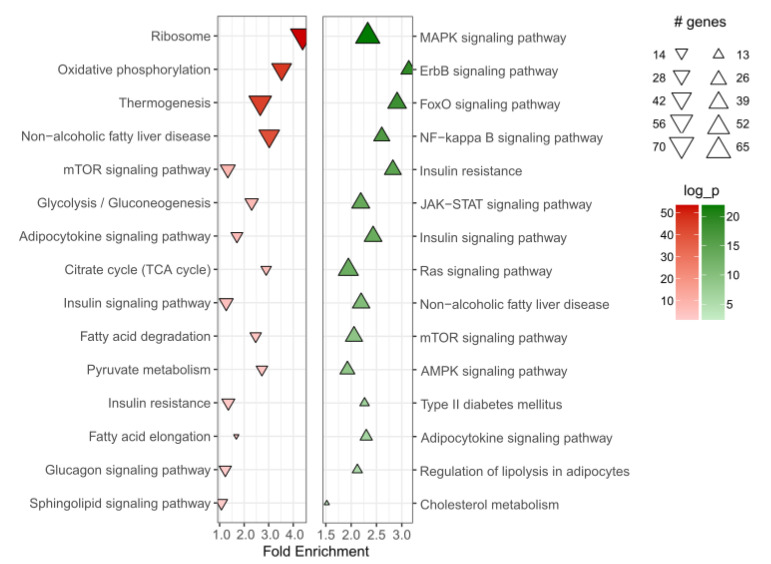
*HMGCR* expression in adipose tissue is associated with the indicated enriched terms identified based on KEGG database. The graph shows a list of positively (green) and negatively (red) associated terms. The complete list of all the enriched terms is provided in Supplementary Figures S1 and S2.

To study the association of *HMGCR* with adipose tissue metabolic pathways, we performed multiple regression analysis between the expression of *HMGCR* and other genes in the adipose tissue from 31 statin-free subjects. The multiple regression analysis was adjusted for BMI, sex, age, and diabetes status. The data showed 7659 genes significantly associated with *HMGCR* expression in adipose tissue. However, after adjusting the regression *p*-values with the Benjamini–Hochberg method, the number of significantly associated genes was 5851. Among these, 2652 genes showed a strong positive association and 3199 genes a strong negative association with *HMGCR* mRNA. The software mapped 2065 positively and 2228 negatively associated genes with the correspondent *p*-values to the protein interaction network and performed an active subnetwork search. After filtering and enriching the active subnetworks with the KEGG gene set, 219 positively associated and 151 negatively associated enriched terms were identified, and among these: metabolic pathways, glucagon and insulin signaling, adipocytokine signaling, oxidative phosphorylation, and citrate cycle. Relevant enriched terms related to metabolism are shown in Figure 1.

Some of the pathways, for instance, insulin signaling, insulin resistance or adipocytokine signaling appear among both positively and negatively associated terms. This is due to the association of *HMGCR* with genes that are involved in different signaling subpathways within the same enriched term. For example, *HMGCR* is positively associated with: *PI3K*, *JNK*, *CD36*, *STAT3*, *ADIPOQ* genes, while it is negatively associated with *mTOR*, *AMPK*, and *PEPCK* genes. All these genes appear in adipocytokine signaling as well as insulin signaling or insulin resistance. A detailed list of genes can be found in Supplementary Table S1.

To understand whether *HMGCR* expression is changed in patients with diabetes, we compared the *HMGCR* adipose tissue gene expression between healthy subjects and individuals with T2D. We did not observe any significant difference in *HMGCR* expression between the two groups. When comparing the expression of *HMGCR* in the adipose tissue of patients taking statins (*n* = 9) vs. statin-free (*n* = 31) individuals, the expression of *HMGCR* was 3-fold higher in the first groups (*p* < 0.05) (data not shown).

We also performed multiple regression analyses of adipose tissue *HMGCR* expression with adipose tissue metabolites. The data showed that *HMGCR* expression correlated with 31 metabolites in adipose tissue. *HMGCR* expression negatively correlated with sphingomyelin. In addition, there was a positive association with phosphatidylserine, total triacylglycerol, and several free fatty acids (e.g., pentadecanoic acid, eicosaenoic acid). However, the association was not significant after multiplicity adjustment with the Benjamini–Hochberg method (data not shown). 

#### 2.1.2. Association of Expression of *HMGCR* and Plasma Metabolites

To investigate the effects of genetic inhibition of *HMGCR* on circulating metabolome, we studied the association of the LDL-lowering *HMGCR* rs12916-T allele, representing genetic inhibition of HMG-CoA reductase, with publicly annotated 174 blood metabolites quantified in large-scale population-based studies [26]. We found a negative association with phosphatidylcholines and sphingomyelins in the blood (Table 1).

#### 2.1.3. Association of *HMGCR* SNP rs12916 with Diabetes and Diabetes-Related Traits

We also studied the association of the T allele of the *HMGCR* polymorphism rs12916 with diabetes and cardiometabolic traits. The results are shown in Table 2. We observed that despite being associated with lower *HMGCR* activity, lower LDL-c levels, and coronary artery disease risk, the rs12916-T allele is associated with a higher risk of type 2 diabetes and several diabetes-associated traits, including increased BMI and body fat % (Table 2).

### 2.2. Observational Study

#### Effects of Statin Treatment on Systemic Insulin Sensitivity

In order to test whether pharmacological inhibition of HMG-CoA reductase would affect systemic insulin sensitivity, we performed an observational study. We compared several metabolic characteristics between 51 patients taking statin therapy and 144 age- and sex-matched individuals in the control group (Table 3). We observed that subjects taking statins show a significant increase in the plasma glucose, HbA1c, insulin levels, as well as HOMA-IR compared to the control subjects (Table 3). We observed no significant difference in BMI, age, and body fat between the mentioned groups. Additionally, the individuals taking statins had significantly lower plasma concentrations of LDL- and total cholesterol compared to the control group, as expected.

After adjustment for age, sex, BMI, HbA1c levels, LDL cholesterol, and diagnosis of diabetes or prediabetes, we found that statin treatment explained 16.8 % of the variability in HOMA-IR (model r^2^ = 0.4969, *p* < 0.01) compared to controls (Table 4).

Having observed that pharmacological inhibition of HMG-CoA reductase with statins was associated with insulin resistance, we aimed to dissect the potential underlying mechanisms and studied the direct effects of simvastatin and its active metabolite on adipose tissue and pancreatic β-cell functions in vitro.

### 2.3. In Vitro Studies

#### 2.3.1. Effects of Simvastatin on Adipocyte Glucose Uptake and Lipolysis

**Figure 2:**
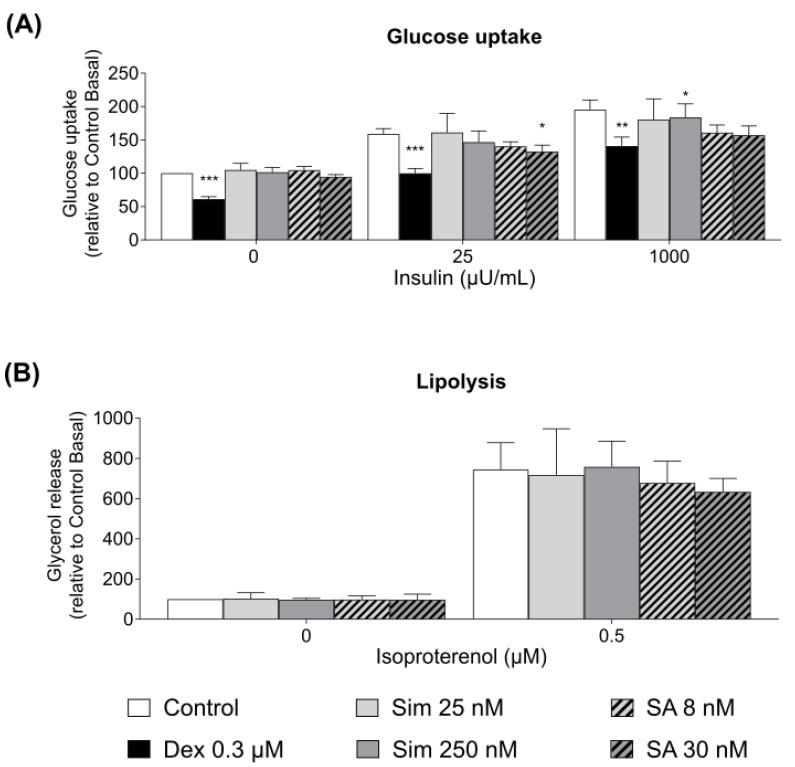
(**A**) Effects of simvastatin and its active metabolite on adipocyte glucose uptake. The graph depicts 14C-glucose uptake by mature human adipocytes isolated from adipose tissue pre-incubated with the given concentrations of simvastatin (*n* = 5–7) and simvastatin hydroxy acid (*n* = 5) for 24 h at basal (0 µU/mL), submaximal (25 µU/mL), and maximal (1000 µU/mL) insulin concentrations. Dexamethasone (Dex, 0.3 µM)—positive control (*n* = 14). (**B**) Effects of simvastatin and its active metabolite on adipocyte lipolysis. The graph shows basal and isoproterenol-stimulated lipolysis measured by glycerol release by mature human adipocytes isolated from adipose tissue pre-incubated with the given concentrations of simvastatin (*n* = 4) and simvastatin hydroxy acid (*n* = 3) for 24 h. Graph shows mean ± S.E.M. * *p* < 0.05, ** *p* < 0.01, and *** *p* < 0.001, relative to Control.

To understand whether statin-associated insulin resistance is relevant to direct effects on adipose tissue, we studied the effects of simvastatin and its active metabolite on human adipocyte glucose and lipid metabolism and differentiation. Incubation of adipose tissue with simvastatin and its active metabolite, simvastatin hydroxy acid, for 24 h has demonstrated no significant effect of therapeutic concentration of the drugs on adipocyte glucose uptake, while the supra-therapeutic concentrations inhibited insulin-stimulated glucose uptake by approximately 10% (*p* < 0.05) (Figure 2A). The viability assay indicated no significant effect of simvastatin and its active metabolite on the viability of adipocytes (data not shown). Dexamethasone was used as a positive control and significantly reduced glucose uptake in adipocytes after 24 h, as expected. Short-term (30 min) incubation of adipocytes with simvastatin did not affect glucose uptake (data not shown).

In addition, simvastatin and simvastatin hydroxy acid had no significant effect on the adipocyte lipolysis after 24 h incubation of the adipose tissue with therapeutic and supra-therapeutic concentrations of the drugs (Figure 2B).

#### 2.3.2. Effects of Simvastatin and Its Active Metabolite on Adipose Tissue Gene Expression

Treatment of adipose tissue with simvastatin and simvastatin hydroxy acid did not affect the mRNA expression of adipokines—adiponectin and leptin, and the pro-inflammatory genes, *IL6* and *IL18*. Therapeutic concentrations of simvastatin and simvastatin hydroxy acid increased mRNA expression of *TFAM* by 30 (*p* < 0.05) and 20%, respectively, but did not affect *PPARGC1A* expression (Table 5). Simvastatin (both 25 and 100 nM) nominally increased *IL1B* (~30–40%).

#### 2.3.3. Effects of Simvastatin on Adipogenesis

**Figure 3:**
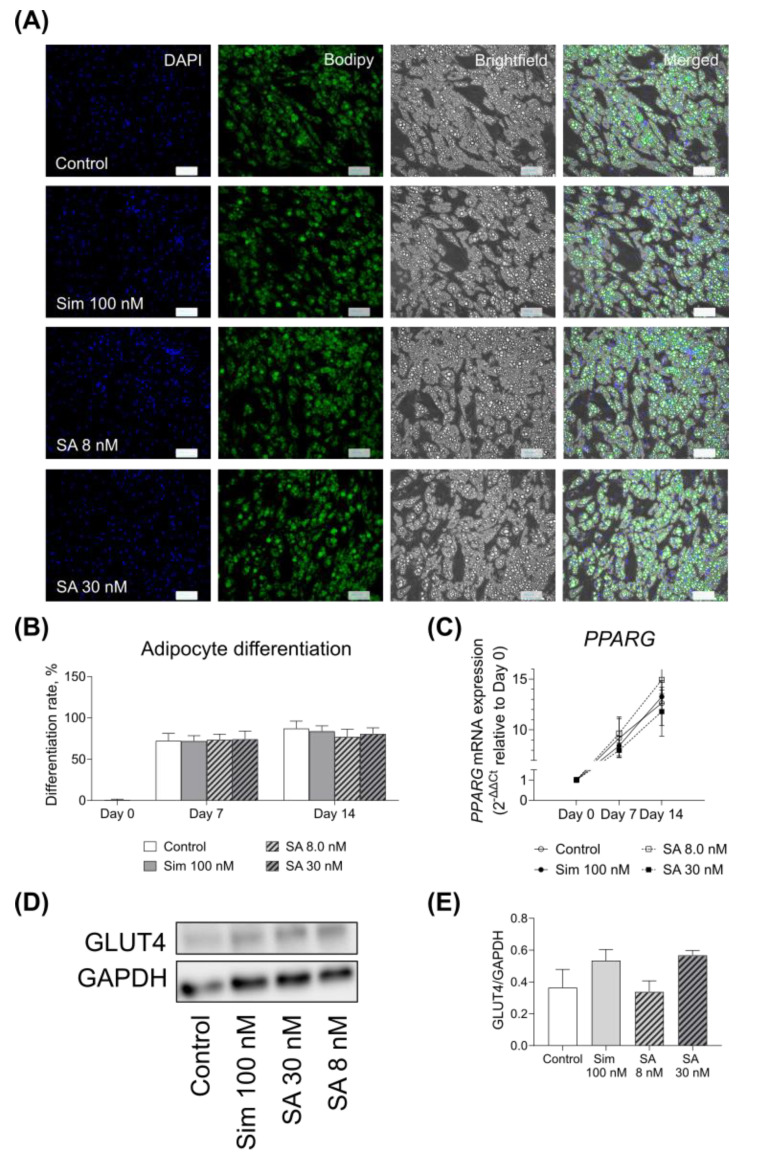
Effects of simvastatin and simvastatin acid on adipocyte differentiation. (**A**) Representative images of differentiation rate of adipocytes treated without (Control) or with Simvastatin 100 nM (Sim 100 nM), simvastatin hydroxy acid 8 and 30 nM (SA 8 nM and SA 30 nM, respectively). Left to right: Nuclei are shown in blue (DAPI), lipid droplets are shown in green (Bodipy), brightfield image, and merged. (**B**) Quantification of adipocyte differentiation rate. (**C**) The differentiation rate was also assessed by measuring the mRNA expression of the master regulator of adipogenesis *PPARG*. *GUSB* was used as the housekeeping gene. Relative gene expression was measured as 2^−ΔΔCt^. (**D**) Representative blot of GLUT4 expression in adipocytes treated without (Control) or with Simvastatin 100 nM (Sim 100 nM), simvastatin hydroxy acid 30 nM and 8.0 nM (SA 30 nM and SA 8 nM), respectively, at day 14. (**E**) Quantification of GLUT4 expression. Graphs show mean ± S.E.M. of *n* = 3 independent experiments.

Treatment of human SGBS cells with simvastatin and its active metabolite during the course of cell differentiation did not affect adipocyte differentiation rate (Figure 3A,B). 

We also measured the gene expression of the master regulator of adipogenesis, peroxisome proliferator-activated receptor gamma (*PPARG*) (Figure 3C). The expression of the gene followed the expected trend increasing over the course of adipocyte differentiation in all the conditions. The data showed that statin treatment during the differentiation did not affect the mRNA expression of *PPARG* (Figure 3C). 

We also observed that Sim 100 nM and both concentrations of SA reduced the expression of *SLC2A4* (GLUT4) mRNA by 30% (*p* < 0.05) and 25% (ns), respectively (Table 6) on day 7 of differentiation. However, this effect disappeared on day 14 of adipocyte differentiation. Similarly, we did not observe any significant change in GLUT4 protein expression in adipocytes after 14 days of differentiation (Figure 3D, E). Sim 100 nM and SA 30 nM reduced the expression of *LEP* and *ADIPOQ* nominally at days 7 and 14 of differentiation. 

Since simvastatin has been shown to induce mitochondrial dysfunction, we also tested its effects on the expression of genes involved in the regulation of mitochondrial function, namely, *PPARGC1A*, *PDK4*, and *TFAM*. We observed no significant effect of the drugs on these genes after 14 days of treatment.

#### 2.3.4. Effects of Simvastatin on β-Cell Function

**Figure 4:**
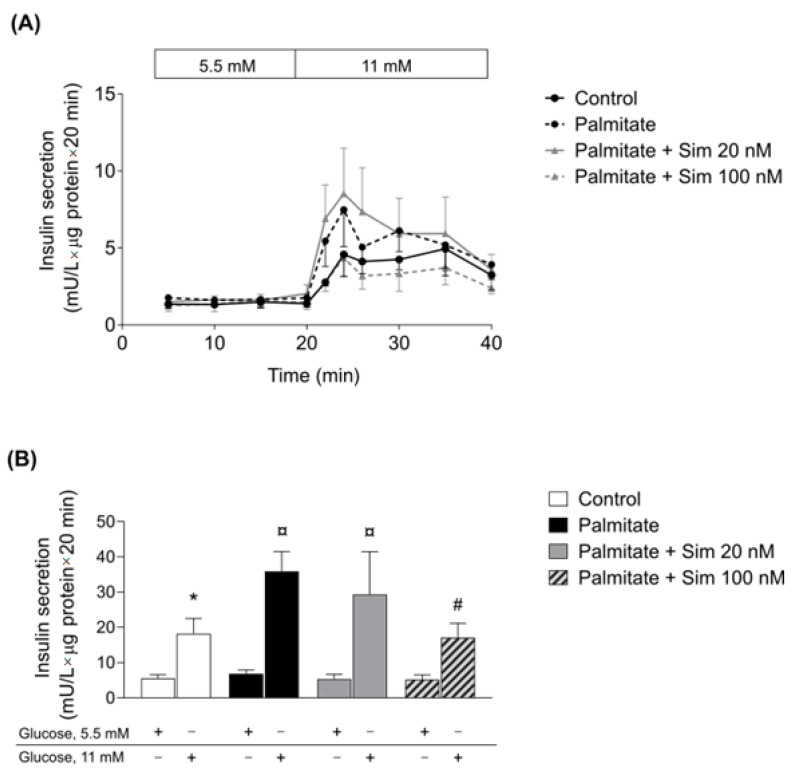
(**A**) Glucose-stimulated insulin secretion from perifused human islets exposed to palmitate (black dashed line), the combination palmitate and simvastatin 20 nM (grey line), the combination palmitate and simvastatin 100 nM (grey dashed line) or in the absence of compounds, control, (black line) for 48 h. Insulin secretion was measured at 5.5 and 11 mmol/L glucose, 20 min each, and normalized to protein. (**B**) AUC insulin secretion for 5.5 and 11 mmol/L glucose was calculated from panel A. Data are shown as mean ± SEM for 4 independent experiments (with 4 different human islet preparations). * *p* < 0.05 compared to control 5.5 mM, ¤ *p* < 0.05 compared to control 11 mM, and # *p* < 0.05 compared to palmitate 11 mM alone (black bar).

Human islets were cultured for 2 days in the presence or absence of 0.5 mM palmitate with or without simvastatin 20 and 100 nM. After culture, dynamic insulin secretion was measured by perifusing islets in low (5.5 mM) and high (11 mM) glucose. Islets treated with palmitate alone for 48 h showed an approximately 2-fold increase in glucose-stimulated insulin secretion (GSIS) compared to control at 11 mM glucose (Figure 4A,B) (*p* < 0.05). At 20 nM, simvastatin did not affect palmitate-induced insulin hypersecretion (Figure 4B); however, simvastatin 100 nM reduced insulin hypersecretion (*p* < 0.05), compared to palmitate treatment. 

## 3. Discussion

In this study, we observed that impaired HMG-CoA reductase activity caused by statin exposure or genetic variants are associated with diabetes and diabetes-related traits. We discovered that *HMGCR* expression is associated with several adipose tissue metabolic pathways, including insulin signaling and citrate cycle, and increased blood circulating levels of phosphatidylcholines and sphingomyelins, as well as insulin resistance. This led us to hypothesize that there might be direct effects of statins on adipose tissue metabolism or β-cell function. Direct treatment of human adipose tissue or β-cells ex vivo modestly reduced adipocyte glucose uptake and β-cell insulin secretion, albeit at supra-therapeutic concentrations. 

Our analysis indicated the involvement of *HMGCR* in a number of metabolic pathways in the adipose tissue. Among these, the most relevant to adipose tissue metabolism include insulin signaling and insulin resistance. Our data showed a positive association of *HMGCR* with insulin signaling pathway, particularly, PI3K signaling, and negative association with NF-κB signaling, which suggests that reduced activity of *HMGCR* could contribute to insulin resistance. A recent study in mice demonstrated that adipose tissue-specific deletion of *Hmgcr* resulted in lipodystrophy and severe glucose intolerance and insulin resistance in the animals [22]. We also observed that *HMGCR* expression in the adipose tissue was also associated with several mitochondrial pathways, including oxidative phosphorylation, citrate cycle, and thermogenesis. Balaz and colleagues have reported the importance of the mevalonate pathway in adipocyte browning and thermogenesis [21]. Furthermore, metabolic side effects of statins were linked to their ability to directly induce mitochondrial dysfunction as well as depletion of mevalonate pathway metabolites [27]. We observed that *HMGCR* expression in the adipose tissue did not differ between healthy individuals and T2D patients. However, the expression of the gene does not represent the activity of the enzyme, therefore, more detailed studies on HMG-CoA reductase are warranted. We compared the expression of *HMGCR* in 31 statin-free individuals with *HMGCR* expression in nine patients taking statins was significantly higher than in the adipose tissue of statin-free subjects. Statin therapy has been shown to induce compensatory upregulation in the expression and activity of HMG-CoA reductase in the liver in animal and human studies [28,29]. It appears that statins have similar effects in human adipose tissue as well. However, these results need to be interpreted with caution as the number of subjects in the two groups was limited and not balanced. 

The T allele of the *HMGCR* polymorphism rs12916 is associated with reduced hepatic *HMGCR* expression and reduced LDL-cholesterol levels [15]. We studied the association of this polymorphism with plasma metabolites and observed a strong negative correlation of the SNP with plasma sphingomyelins and phosphatidylcholines. The plasma concentrations of phosphatidylcholines and sphingomyelins were reduced in subjects with impaired HMG-CoA reductase activity. Reduced HMG-CoA reductase activity leads to diminished conversion of HMG-CoA to mevalonate [1]. Mevalonate has been reported to increase sphingomyelin levels via inhibiting acid sphingomyelinase activity [30], and therefore reduced sphingomyelin levels in subjects with reduced HMG-CoA reductase activity would have been expected. Inhibition of the HMG-CoA reductase has been shown to inhibit de novo synthesis of phosphatidylcholines via the cytidine diphosphate-choline pathway, which, in turn, could reduce its plasma lipid levels [31]. Although most clinical studies suggested that sphingomyelins and phosphatidylcholines, such as C32:2, C34:1, and C34:2, are associated with reduced insulin sensitivity in humans [32,33], a metabolomic analysis in newly developed type 2 diabetes subjects shows that some phosphatidylcholines, are significantly associated with decreased risk of diabetes [33,34]. Interestingly, we observed a negative association of reduced *HMGCR* activity with plasma sphingomyelins and phosphatidylcholines, suggesting that any disruption of phospholipid metabolism could affect metabolism. However, whether changes in phospholipids are a cause or consequence of insulin resistance requires further investigation. 

Since pharmacological inhibition of HMG-CoA reductase with statins is associated with new-onset diabetes in patients, we compared insulin resistance between subjects taking statins and age- and BMI-matched control subjects. We tested the effect of statin treatment on systemic glucose tolerance in another independent cohort of 195 individuals, to verify whether the diabetogenic effect of statins might be due to pathophysiological mechanisms occurring in other tissues. After adjusting for multiple confounders, including BMI, age, sex, diabetes status, HbA1c, and LDL cholesterol levels, statins use was associated with an increased HOMA-IR. Similarly, diabetogenic effects of statins have been reported in patients previously [6,8,35]. 

Since statin therapy was associated with increased systemic insulin resistance, we hypothesized that the diabetogenic effects of statin treatment could be mediated via a direct effect on adipose tissue and β-cell function. We observed that the drug or its active metabolite led to a 10% reduction in insulin-stimulated adipocyte glucose uptake only at the supra-therapeutic concentrations. Our data are consistent with the study on 3T3-L1 adipocytes treated with 100 ng/mL of simvastatin (ca 230 nM), which slightly reduce adipocyte glucose uptake [23]. To the best of our knowledge, the effects of simvastatin hydroxy acid on adipocyte glucose uptake have not been previously studied. A similar trend in glucose uptake regulation was expected, as both drugs target the same enzyme. However, adipose tissue accounts for less than 10% of the whole-body glucose uptake [35]. Therefore, the modest 10% reduction of the adipocyte glucose uptake is unlikely to lead to an increased risk of type 2 diabetes, as observed in patients taking statins. Neither simvastatin nor its active metabolite altered adipocyte lipolysis after 24 h incubation of the adipose tissue. Our data are in accordance with the study conducted by Henriksbo and colleagues, who reported no effects of atorvastatin on 3T3-L1 adipocyte lipolysis and glucose uptake [35]. 

The presence of simvastatin and its active metabolite during the adipogenesis of SGBS cells did not alter their differentiation rate. Similar to our findings, Nakata and colleagues did not observe any changes in murine 3T3-L1 adipocyte maturation with simvastatin [23]. We did not observe any significant effect on adipocyte GLUT4 expression after 14 days of differentiation at both mRNA and protein levels, while Nakata and co-workers reported that simvastatin reduced GLUT4 expression in 3T3-L1 cells after 10 days of culture [23]. The discrepancy in our data could be due to the differences in the experimental setup, such as different cell lines or concentration differences, as Nakata and colleagues used a much higher concentration of the drug than in our setting.

We observed no significant effect of simvastatin and its active metabolite, SA, on the mRNA expression of adipokines in the adipose tissue. It has been shown that gene expression of adiponectin was not altered in the adipose tissue of animals treated with simvastatin for 2 weeks, while leptin mRNA expression was increased by the statins [36]. Maeda and Horiuchi reported a reduction in the expression of leptin mRNA in 3T3-L1 adipocytes differentiated in the presence of simvastatin [24]. Our data similarly showed a nominal reduction in *LEP* and *ADIPOQ* expression in SGBS cells. Similarly, reduced serum adiponectin concentration was reported in patients treated with simvastatin for 2 months [37]. 

Simvastatin treatment nominally increased the expression of *IL1B* in the adipose tissue. Our data are in accordance with the study by Henriksbo and colleagues, who reported that statins, including simvastatin, induce IL1β secretion via NLRP3 inflammasome activation in macrophages, which are the dominant leukocyte type in the adipose tissue [38,39]. Although statins have been reported to have anti-inflammatory effects by reducing circulating C-reactive protein and pro-inflammatory cytokines, they appear to induce adipose tissue inflammation. Interestingly, our data did not show any significant effect of simvastatin on the genes involved in the regulation of mitochondrial functions, namely *PPARGC1A*, *TFAM*, and *PDK4*. We also did not observe any changes in *CAV1* expression, which could be used as a marker of cholesterol depletion in adipose tissue [36]. This could be due to a relatively short-term incubation of adipose tissue and adipocytes with the drugs, while the patients taking statins are exposed to the drug for a much longer period. Additionally, the majority of in vitro studies use high concentrations of the drug [25], while we selected the concentrations relevant to the plasma levels of the drugs. 

Although our study demonstrated no direct effects of simvastatin and its active metabolite on adipose tissue metabolism, genetic or pharmacological inhibition of HMG-CoA reductase has been associated with weight gain, which itself is a risk factor for the development of diabetes [15,40] therefore, increased adiposity could be one of the contributors to diabetogenic effects of statins.

Similar to adipose tissue, we did not observe a significant effect of therapeutic concentration of simvastatin on the human pancreatic islets. When we increased the simvastatin concentration, the statin normalized palmitate-induced insulin hypersecretion, which has been suggested as a treatment strategy for early obesity intervention [41]. In human islets exposed to a high statin concentration alone, GSIS was reduced [42]. Thus, to what extent the observed positive effect of the higher statin concentration in β-cells of the palmitate-treated islets in the present study could be of clinical use remains to be decided. Statin-induced reduction in insulin secretion from β-cells happens because statins disrupt the production of endogenous cholesterol in β-cells, which is important to maintain the functionality of CaV channels and insulin secretion [17]. Additionally, pancreatic β-cell-specific ablation of *HMGCR* gene in mice resulted in a phenotype with severe hypoinsulinemia and hyperglycemia [43]. This study revealed the importance of *HMGCR* and mevalonate pathway for pancreatic β-cell development [43]. 

Our study has several limitations. One of the limitations is the short incubation time compared to the duration of the treatment in the patients. HMG-CoA reductase is highly important for adipose tissue and pancreas functions [22,43]. Although 24 h incubation of MIN6 cells with atorvastatin (10 µM) has shown a reduction in cholesterol content in the cells [44], measuring the cholesterol content or *HMGCR* activity in human islets and adipose tissue is warranted. Unfortunately, it was not possible in our setting due to the limited amount of material and reduction in the number of donors because of the COVID-19 pandemic. To our best knowledge, there are no reports on the pancreatic β-cell function in individuals with rs12916 T-allele, which would be an important asset to understanding the role of HMG-CoA reductase in islet function. Additionally, glucose-stimulated insulin secretion in the human islets is also warranted, as we only measured GSIS after treatment of pancreatic islets with palmitate, which is a diabetogenic condition [45]. Another limitation is a small sample size for both adipose tissue and pancreatic islet donors. Finally, for the observational study results, although we tried to adjust for several confounding factors, these data need to be interpreted with caution as the observational study performed in this work had a selection bias since we can assume that patients on statin treatment display a less favorable metabolic profile and are more prone to develop metabolic syndrome. In addition, it should be noted that there could be more parameters to consider, such as treatment duration, the dose of the medication, as well as the age of the patients since high doses of statins, older age, and female sex were associated with a higher risk of new-onset diabetes [8,46,47]. 

Our study targeted the effects of statins on human cells and tissues. Our data show that genetic inhibition of HMG-CoA reductase is negatively correlated with plasma sphingomyelins and phosphatidylcholines, which appear to play a role in insulin resistance development. Our results suggest that *HMGCR* expression and activity in adipose tissue are associated with metabolic pathways; however, statin treatment has a minor direct effect on adipose tissue metabolism and β-cell insulin secretion. Other tissues, such as liver or skeletal muscle, might be of greater interest when investigating the diabetogenic effects of statins. 

## 4. Materials and Methods

### 4.1. Subjects and Adipose Tissue Biopsies

Overall, we included 216 individuals of whom 51 were on statin treatment (33—simvastatin; 17—atorvastatin; 1—rosuvastatin) into the study. Subjects fasted overnight (>10 h), and fasting venous samples were collected for biochemical analysis of glucose, insulin, HbA1c, and lipids at the Department of Clinical Chemistry, Uppsala University Hospital. Anthropometric characteristics, fasting blood biochemical values, and T2D status of the subjects are shown in Table 7. Additionally, pancreatic islets were collected from 4 brain-dead non-diabetic donors (3M/1F, Age: 19–27, BMI: 19.7–27.5 kg/m^2^).

Individuals with type 1 diabetes mellitus and other diseases, such as cancer or endocrine disorders, were excluded. The study was approved by the Swedish Ethical Review Authority in Uppsala (DNR 2013/183–494 and 2018/385), and all participants gave their written informed consent. Ethical permission to use human islets isolated from human donors has been obtained from the Regional Ethical Review Board in Uppsala (EPN number 2010/006).

Needle biopsies were obtained from subcutaneous abdominal adipose tissue from 43 healthy subjects (17M/26F, Age: 19–72 yo, BMI: 21.8–38.4 kg/m^2^) and 20 individuals with T2D (10M/10F, Age: 41–71, BMI: 22.4–39.9 kg/m^2^) with local anesthesia with lidocaine (Xylocain, AstraZeneca, Södertälje, Sweden). The anthropometric and biochemical characteristics of the T2D subjects have been previously reported by Pereira and colleagues [48]. Due to the limited amount of adipose tissue obtained, not all experiments were performed in all biopsies. Adipose tissue was used for transcriptomics and metabolomics, and association studies (*n* = 31, see Section 4.2). In addition, adipose tissue was used for in vitro incubation with simvastatin to study the effects on glucose uptake (*n* = 14), lipolysis (*n* = 7), and adipose tissue gene expression (*n* = 9) (see Section 4.4). 

Our study consists of three parts, for which the indicated number of subjects was recruited:

### 4.2. Association Studies

Transcriptomics and metabolomics data from 31 statin-free subjects were used to study the association of the gene expression of *HMGCR* in adipose tissue with other adipose tissue genes and adipose tissue and plasma metabolome. This group also included healthy individuals as well as patients with T2D. The subjects with T2D were on monotherapy with a stable metformin dose (mean metformin dose 500 mg).

#### 4.2.1. Association Studies of Adipose Tissue *HMGCR* Gene Expression

*HMGCR* expression in adipose tissue was correlated with other adipose tissue genes and adipose tissue and plasma metabolome of 31 statin-free subjects. This group also included healthy individuals as well as patients with T2D. The analysis was performed using multiple regression analysis adjusted for subjects’ BMI, sex, age, and diabetes status with Statsmodel [46] and SciPy [47] packages for Python 3.8.6. *p*-value was adjusted with Benjamini–Hochberg correction. The list of significantly correlated genes was processed using the pathfindr.R package, which allows mapping of major biochemical pathways associated with the genes obtained [49]. The full list of all the pathways is available upon request. 

#### 4.2.2. *HMGCR* SNPs Analysis

We have used publicly available data from PhenoScanner (http://www.phenoscanner.medschl.cam.ac.uk/, accessed on 23 March 2021) [50,51] to assess the association of rs12916 T-allele SNPs in *HMGCR* region with diabetes or diabetes-related traits. SNP position is chr5:74656539. We obtained a list of 11 traits; hence, all the *p*-values were adjusted with Bonferroni correction with the factor of 11.

#### 4.2.3. Impact of a Genetic *HMGCR* Variant Lowering LDL Levels on the Circulating Metabolome

We accessed genetic association results from publicly available data from a GWAS study of circulating levels of 174 blood metabolites [26] for *HMGCR* variant rs12916, which has previously been linked to lower blood LDL cholesterol [15]. We corrected the *p*-values using the Benjamini–Hochberg method. 

### 4.3. Observational Study

The effect of statin treatment on the HOMA-IR index, i.e., an index of insulin resistance, was assessed in 195 individuals (51 individuals on statin therapy and 144 age-, sex-, BMI-matched controls) (Table 3). The cohort included healthy subjects as well as patients diagnosed with T2D or prediabetes.

We performed linear regression analysis with adjustment for age, sex, BMI, HbA1c levels, LDL cholesterol, and diagnosis of diabetes or prediabetes. Continuous variables were log-transformed.

### 4.4. In Vitro Studies

#### 4.4.1. Isolation of Human Mature Adipocytes

Mature adipocytes were isolated from adipose tissue digested for 1 h at 37 °C and 105 RPM with 1.2 mg/mL of collagenase A (Clostridium histolyticum, Roche, Manheim, Germany) in Medium 199 (Gibco, Life Technologies, Paisley, UK) complemented with 4% bovine serum albumin (BSA, Sigma, St.Louis, MO, USA), 6 mM glucose, and 150 nM adenosine (Sigma, St. Louis, MO, USA), with pH adjusted to 7.4 with 1M NaOH (Sigma, St. Louis, MO, USA). Isolated adipocytes were filtered through 250 µm nylon mesh and further used for ex vivo lipolysis and glucose uptake. For lipolysis, isolated adipocytes were washed and diluted with Medium 199 to 3–5% lipocrit. Glucose uptake experiments were performed with glucose-free Krebs-Ringer-Hepes medium (KRH), supplemented with 4% BSA, 150 nM adenosine, and pH 7.4. Isolated adipocytes were washed with KRH and diluted to 6–7% lipocrit. Average adipocyte diameter was measured consecutively in 100 adipocytes and the number of cells in the incubation samples as reported previously [52].

#### 4.4.2. Adipocyte and Adipose Tissue Incubation

In order to reflect the clinical conditions, the concentration of the drugs for the study was selected according to the reported therapeutic plasma concentrations of simvastatin (25 nM) and its active metabolite simvastatin hydroxy acid (8 nM) [25,53,54]. We also used a concentration four times higher to cover all relevant therapeutic concentrations.

Subcutaneous adipose tissue obtained from needle biopsies was incubated in 6 mM glucose Dulbecco’s Modified Eagle Medium (DMEM), supplemented with 10% fetal bovine serum (FBS) and 1% penicillin-streptomycin without (Control) or with simvastatin (25 nM and 100 nM) and its active metabolite simvastatin hydroxy acid (8 nM and 30 nM) for 24 h (*n* = 16). Synthetic glucocorticoid dexamethasone (Dex) was used as a positive control, as it has been reported to reduce the adipose tissue glucose uptake [55,56]. After the incubation with the drugs, adipose tissue was digested as described above, and mature adipocytes were isolated for adipocyte glucose uptake and lipolysis measurement. Adipose tissue after the incubation was also washed with ice-cold phosphate-buffered saline (PBS, Medicago, Uppsala, Sweden) and was snap-frozen and transferred to –80 °C for further gene and protein expression analysis. Due to the limited amount of adipose tissue obtained per biopsy, not all experiments could be performed in the same tissue from all subjects.

#### 4.4.3. Statins Effect on Adipocyte Glucose Uptake

Glucose uptake assessment was performed in adipocytes after incubating adipose tissue with simvastatin as described previously [57]. In short, following the incubation with the drugs, isolated adipocytes were washed with glucose-free KRH medium (4% BSA, 150 nM adenosine, pH = 7.4). Adipocytes were diluted with KRH to 6–7% lipocrit. The lipocrit was transferred to vials kept in a water bath at 37 °C, shaking at 65 RPM. The cells were further stimulated for 15 min without or with physiological and supra-physiological concentrations of insulin (25 µU/mL and 1000 µU/mL, Actrapid, NovoNordisk, Bagsvaerd, Denmark). Following the insulin stimulation, adipocytes were incubated for 45 min with 0.26 mCi/L (0.86 µM) D-[U-14C]-glucose (Perkin Elmer, Boston, MA, USA). The reaction was terminated via transferring the cell suspension to pre-cooled vials on ice. To allow the separation of adipocyte cell pellet from the medium, the lipocrit was further centrifuged through 1 mL of silicone fluid 100 cS (VWR Chemicals, Leuven, Belgium). After that, Ultima Gold scintillation cocktail (Perkin Elmer, Walthman, MA, USA) was added to the cell pellet, and the cellular glucose uptake was calculated as described previously [53], according to the following calculations:

(1) $$\mathrm{Cell}\;\mathrm{clearance}\;\mathrm{of}\;\mathrm{medium}\;\mathrm{glucose}=\frac{\mathrm{cell-associated}\;\mathrm{radioactivity}\;\times \;\mathrm{volume}}{\mathrm{radioactivity}\;\mathrm{of}\;\mathrm{the}\;\mathrm{medium}\;\times \;\mathrm{cell}\;\mathrm{number}\;\times \;\mathrm{time}}$$

Cell-associated radioactivity was measured with Liquid Scintillation Analyser Tri-Carb 4910 TR (Perkin Elmer, Boston, MA, USA). Glucose uptake was calculated per cell number, and data were normalized to the respective control in each condition. 

#### 4.4.4. Statin Effects on Adipocyte Lipolysis

After the pre-incubation of adipocytes or adipose tissue with simvastatin, isolated adipocytes were diluted at a lipocrit of 2–3% with Medium 199 (6 mM glucose, 4% BSA, 150 nM adenosine, pH 7.4). The cell suspension was transferred to vials in a water bath at 37 °C, shaking at 65 RPM. Adipocytes were stimulated for 10 min with 0–100 IU/mL insulin, 37 °C, 65 RPM. Cells were further co-incubated for 2 h at 37 °C without or with 0.5 µM β-receptor agonist isoproterenol (Sigma). The incubation was stopped by transferring the vials on ice. Basal and isoproterenol-stimulated glycerol release was measured as an indicator of lipolysis rate. The glycerol release was detected via colorimetric absorbance at 540 nm with Free Glycerol Reagent (Sigma) with SpectraMax iD3 (Molecular Devices, San Jose, CA, USA). Glycerol release was normalized to cell number, and all the experiments were performed in triplicates.

#### 4.4.5. Effect of Statins on Adipocyte Differentiation

Human Simpson–Golabi–Behmel syndrome (SGBS) adipocytes were kindly provided by Professor Martin Wabitsch (Ulm University Medical Centre, Ulm, Germany). SGBS at passage 3 were expanded in DMEM-F12 supplemented with 33 µM biotin (Sigma, St Louis, MO, USA), 17 µM pantothenate (Sigma, St Louis, MO, USA), 1% PEST (Gibco, Life Technologies, Paisley, UK), and 10% non-heat-inactivated FBS (Gibco, Life Technologies, Paisley, UK). At 80% confluence, the cells were detached with trypsin (Gibco, Life Technologies, Paisley, UK) and seeded in 12-well plates (Sarstedt, Nümbrecht, Germany) at density 30,000 cells/well. After reaching 90% confluence, adipocyte differentiation was induced for 4 days with differentiation medium: DMEM-F12 with 1% PEST (Gibco, Life Technologies, Paisley, UK), 100 nM insulin, 17 µM pantothenate, 33 µM biotin, 1 µM dexamethasone, 1 µM rosiglitazone, 250 µM 3-isobutyl-1-methylxanthine, 10 µg/mL transferrin, 2 nM tri-iodothyronine (T3) (all from Sigma, St Louis, MO, USA). The cell line handling was performed according to the previously reported protocol [54]

The differentiation medium was replaced with a maintenance medium (the same composition as the differentiation medium except for IBMX, dexamethasone, and rosiglitazone) on day 5. The adipocyte differentiation was sustained until day 14. The medium was changed every 2 days. The differentiation of adipocytes was estimated on days 0, 7, and 14 post-induction via measuring the expression of adipogenic markers. *GUSB* was selected as a stable housekeeping gene for gene expression analysis.

In order to assess the adipocyte differentiation rate, on differentiation day 14, the cells were washed with PBS and fixed in 4% formaldehyde (Histolab, Gothenburg, Sweden) for 15–20 min at RT. The cells were further stained with Hoechst 33342 in PBS and Bodipy 493/503 (4,4-Difluoro-1,3,5,7,8-Pentamethyl-4-Bora-3a,4a-Diaza-s-Indacene; Molecular Probes, OR, USA) dyes for 20 min at RT for staining the nuclei and lipids, respectively. The images were acquired with ImageXpress Pico Automated Cell Imaging System (Molecular Devices, San Jose, CA, USA). Adipocyte differentiation rate was assessed at differentiation days 7 and 14 by quantifying the number of cells positive for GFP signal from the Bodipy-stained lipids, normalized to total cell number. 

#### 4.4.6. Statin Effects on Adipose Tissue and Adipocyte Gene Expression

Adipose tissue or adipocytes treated without or with simvastatin was snap-frozen and stored at −80 °C for gene expression analysis. RNA extraction was performed with phenol/chloroform extraction method. In brief, adipose tissue or adipocytes were lysed with Trizol Reagent (ThermoFisher, Carlsbad, CA, USA) followed by the addition of chloroform to the homogenate. The aqueous phase was further transferred to isopropanol (Sigma) and incubated to allow RNA precipitation. The pellet was washed three times with 70% ethanol (Solveco, Roserberg, Sweden) [58]. The RNA was eluted, and its concentration and quality were measured with NanoDrop 2000 spectrophotometer (ThermoFisher Scientific, Rockford, IL, USA). Complementary DNA (cDNA) was reverse-transcribed from 400 ng of RNA with High-Capacity cDNA Reverse Transcription Kit (Applied Biosystems, ThermoFisher Scientific, Foster City, CA, USA). The gene expression analysis was performed with QuantStudio 3 System (Applied Biosystems, ThermoFisher Scientific, Waltham, MA, USA). The Taqman probes (ThermoFisher Scientific) were used for: *ADIPOQ*, *CAV1*, *IL18*, *IL1B*, *IL6*, *LPL*, *LEP*, *PDK4*, *PPARG*, *PPARGC1A*, *SLC2A4*, and *TFAM*. Relative gene expression was normalized to housekeeping gene *18S* ribosomal RNA (18S rRNA) or *GUSB* expression. All samples were run in duplicates, and the gene expression was calculated as 2^−ΔΔCt^ relative to Control, where ΔΔCt = ΔCt_sample_ − ΔCt_control_ and ΔCt = Ct_target gene_ − Ct_housekeeping gene_. 

#### 4.4.7. Western Blot

Preadipocytes were collected on days 7 and 14 of differentiation, and total protein was isolated with lysis buffer (25 mM Tris-HCl; 0.5 mM EGTA; 25 mM NaCl; 1% Nonidet P-40; 1 mM Na_3_VO_4_; 10 mM NaF (all from Sigma, St Louis, MO, USA); 100 nM okadaic acid (Alexis Biochemicals, Lausen, Switzerland), 1X Complete protease inhibitor cocktail (Roche, Indianapolis, IN, USA), and pH 7.4). The protein concentration of the lysates was measured using a BCA protein assay kit (Pierce, Thermo Scientific, Rockford, IL, USA).

Protein lysates (8–10 µg) were loaded and separated by SDS-PAGE (10% Mini-Protean TGX Stain-free gels, BioRad, Hercules, CA, USA). This was followed by transfer to nitrocellulose membranes and blocked with 5% non-fat milk (Sigma) in 0.05% tween-phosphate buffer saline (PBST, Medicago, Uppsala, Sweden) for 1 h at room temperature. Membranes were incubated overnight with the primary antibody anti-GLUT4 (1:1000, MA1–83191, Invitrogen, ThermoFisher Scientific, Waltham, MA, USA). Glyceraldehyde-3-phosphate dehydrogenase was detected with anti-GAPDH (1:2000, Millipore, Temecula, CA, USA) was chosen as a loading control protein. After incubation with primary antibody, the membranes were washed with PBST and incubated with horseradish peroxidase-conjugated anti-rabbit or anti-mouse (Cell Signaling Technologies, Danvers, MA, USA) secondary antibodies, respectively. Protein bands visualization was done using enhanced chemiluminescence with a high-resolution field and quantified with ChemiDocTM MP System (BioRad, Hercules, CA, USA).

#### 4.4.8. Incubation of Human Islets and Insulin Determination

Human islets were supplied by the Islet Transplantation Unit (Department of Radiology, Oncology and Clinical Immunology, Uppsala University Hospital, Uppsala, Sweden) and cultured in CMRL 1066 medium (Invitrogen, Paisley, UK) containing 5.5 mM glucose, 10% fetal bovine serum, 1% penicillin-streptomycin and 1% glutamine (Invitrogen) at 37 °C and 5% CO_2_. Palmitate was prepared as described previously [59]. Human islets were treated with or without 0.5 mM palmitate in the presence or absence of either 20 nM or 100 nM simvastatin (Sigma, St Louis, MO, USA) for 48 h.

Insulin secretion from human islets was measured as described previously [59]. In brief, the islets were perifused in KRBH buffer (130 mmol/L NaCl, 4.8 mmol/L KCl, 1.2 mmol/L MgSO_4_, 1.2 mmol/L KH_2_PO_4_, 1.2 mmol/L CaCl_2_, 5 mmol/L NaHCO_3_, 5 mmol/L HEPES, pH 7.4) supplemented with 1 mg/mL BSA and 5.5 mM glucose at 37 °C for 1 h. Following the initial perifusion, aliquots of the medium were collected every 5 min for 20 min at 5.5 mM glucose. After this, the medium was replaced with 11 mM glucose and the samples were obtained at 1, 2, and 5 min for the next 20 min. Insulin secretion was measured with enzyme-linked immunosorbent assay (ELISA) as described previously [59].

### 4.5. Statistical Analysis

All data represent mean ± SEM unless stated otherwise. Transcriptomics, metabolomics, and clinical data analyses were performed using multiple regression analysis with multiple comparison *p*-value adjustment with the Benjamini–Hochberg method. Comparison of the means of more than two groups was performed with repeated-measures One-Way ANOVA test with multiple comparison *p*-value adjustment with False Discovery Rate correction. * (*p* < 0.05), ** (*p* < 0.01), *** (*p* < 0.001). The means of the two groups were compared using an unpaired parametric or non-parametric test, depending on data distribution. Analyses were performed with Statsmodel 0.5.0 [46] and SciPy 1.7.0 [47] packages for Python 3.8, and GraphPad Prism 9.0 software. Gene enrichment analysis was performed with pathfindr.R package [49]. Data visualization was performed with GraphPad Prism, pathfindr.R package [49] and Inkscape software.

## Figures and Tables

**Table 1:** List of metabolites in the blood associated with *HMGCR* rs12916-T allele.

Metabolite	Metabolite Class	*Z* Score	*p*-Value	Adj. *p*-Value
PC aa C36:3	Phosphatidylcholines	−3.236	0.0012	0.0014
SM C24:0	Sphingomyelins	−3.24	0.0012	0.0029
PC ae C34:2	Phosphatidylcholines	−3.13	0.0017	0.0043
PC aa C36:2	Phosphatidylcholines	−3.12	0.0018	0.0057
PC aa C34:3	Phosphatidylcholines	−3.067	0.0022	0.0072
PC ae C40:4	Phosphatidylcholines	−3.057	0.0022	0.0086
PC aa C32:3	Phosphatidylcholines	−2.956	0.0031	0.0101
PC ae C32:2	Phosphatidylcholines	−2.955	0.0031	0.0115
PC aa C26:0	Phosphatidylcholines	−2.873	0.0041	0.0129
PC ae C34:1	Phosphatidylcholines	−2.828	0.0047	0.0144
Hydroxyhexadecanoylcarnitine	Acylcarnitines	−2.809	0.005	0.0158
PC ae C36:2	Phosphatidylcholines	−2.807	0.005	0.0172
PC aa C28:1	Phosphatidylcholines	−2.766	0.0057	0.0187
PC ae C44:3	Phosphatidylcholines	−2.69	0.0072	0.0201
PC aa C34:1	Phosphatidylcholines	−2.676	0.0074	0.0216
Octadecanoylcarnitine	Acylcarnitines	−2.614	0.009	0.0230
PC aa C30:0	Phosphatidylcholines	−2.599	0.0094	0.0244
Asparagine	Amino acids	2.594	0.0095	0.0259
Dodecanoylcarnitine	Acylcarnitines	−2.578	0.0099	0.0273

PC aa—Phosphatidylcholine with diacyl residue; PC ae—Phosphatidylcholine with acyl-alkyl residue; SM—sphingomyelin.

**Table 2:** Association of the T-allele of rs12916 (Chr. 5, position: 74656539) with diabetes and diabetes-related traits in large published GWAS.

Trait	Unit	Study PMID	Beta	*p*-Value	Adj.*p*-Value	*n*
2 h fasting glucose	mmol/L	22,885,924	0.02	0.14	1	42,854
Body fat percentage	IVNT	UKBB *	0.01	1.4 × 10^−9^	1.6 × 10^−8^	331,117
Body mass index	IVNT	25,673,413	0.02	7.3 × 10^−9^	8.0 × 10^−8^	315,585
Coronary artery disease	log OR	28,530,674	−0.04	7.6 × 10^−6^	8.4 × 10^−5^	214,613
Fasting glucose	mmol/L	22,885,924	0.00	0.86	1	133,010
High density lipoprotein	IVNT	24,097,068	0.00	0.20	1	187,167
Hip circumference	IVNT	25,673,412	0.02	2.80 × 10^−5^	3.1 × 10^−4^	213,038
log Fasting insulin	pmol/L	22,885,924	0.01	4.72 × 10^−3^	0.05	108,557
LDL cholesterol	IVNT	24,097,068	−0.07	7.8 × 10^−78^	8.6 × 10^−77^	168,357
Type 2 diabetes	logOR	25,262,344	0.06	9.60 × 10^−5^	1.1 × 10^−3^	89,371
Waist circumference	IVNT	25,673,412	0.02	9.70 × 10^−7^	1.1 × 10^−5^	232,101

* http://www.nealelab.is/blog/2017/7/19/rapid-gwas-of-thousands-of-phenotypes-for-337000-samples-in-the-uk-biobank (accessed on 18 August 2021).

**Table 3:** Anthropometric and fasting biochemical characteristics of healthy subjects (Control) and statin patients (Statins) involved in the study.

Variable	Control	Statins	*p*-Value
*n*	144	51	
M/F	49/95	18/33	
Age	59.0 ± 10.5	62.2 ± 7.7	0.1094
BMI	30.4 ± 6.1	30.5 ± 5.6	0.8208
Body fat, %	38.5 ± 10.1	38.9 ± 9.0	0.9573
Glucose, mmol/L	6.3 ± 1.3	7.2 ± 1.7	<0.001
HbA1C, mmol/mol	38.4 ± 8.0	44.5 ± 11.3	<0.001
Insulin, mmol/L	11.4 ± 7.9	15.4 ± 11.8	0.0250
HOMA-IR, mmol × µU/L^2^	3.1 ± 2.3	4.9 ± 4.1	0.0021
Cholesterol, mmol/L	4.6 ± 2.0	4.1 ± 1.5	0.0060
HDL cholesterol, mmol/L	1.9 ± 1.0	1.5 ± 0.6	0.0162
LDL cholesterol, mmol/L	3.4 ± 0.9	2.6 ± 0.7	<0.001
Triglycerides, mmol/L	1.3 ± 0.6	1.6 ± 0.8	0.0113

Statistical analysis was performed with a non-parametric Mann–Whitney test for unpaired samples. *n*—number; HbA1c—glycosylated haemoglobin; HOMA-IR—homeostatic model assessment of insulin resistance, HDL—high-density lipoprotein; LDL—low-density lipoprotein. The data are shown as mean ± SD.

**Table 4:** Linear regression analysis for estimating the impact of statin treatment and LDL-cholesterol on HOMA-IR. All continuous variables and the outcome were log-transformed.

Variable	Std. β	*p*-Value
Statin	0.168	0.006
BMI	0.415	0.000
HbA1c	0.215	0.014
Age	−0.031	0.605
Sex	−0.119	0.030
Diabetes	0.233	0.008
LDL cholesterol	0.154	0.008

HbA1c—glycosylated haemoglobin; LDL—low-density lipoprotein.

**Table 5:** Fold-change in gene expression in adipose tissue after 24 h incubation with simvastatin (Sim) and simvastatin acid (SA).

Gene Symbol	Sim 25 nM	Sim 100 nM	SA 8 nM	SA 30 nM	Dex 0.3 µM
	Fold Change	Fold Change	Fold Change	Fold Change	Fold Change
Adipokines					
*ADIPOQ*	1.11 ± 0.07	1.08 ± 0.13	1.12 ± 0.13	1.02 ± 0.11	0.91 ± 0.06
*LEP*	1.02 ± 0.05	1.01 ± 0.07	1.18 ± 0.15	0.97 ± 0.08	1.64 ± 0.39
Pro-inflammatory cytokines					
*IL6*	1.10 ± 0.20	1.22 ± 0.22	1.37 ± 0.24	1.05 ± 0.16	0.20 ± 0.04 *
*IL1B*	1.31 ± 0.23	1.42 ± 0.19	1.19 ± 0.14	1.15 ± 0.14	0.14 ± 0.05 *
*IL18*	1.03 ± 0.07	1.01 ± 0.06	1.03 ± 0.09	1.00 ± 0.09	3.23 ± 0.54 *
Regulation of mitochondrial function					
*PPARGC1A*	1.08 ± 0.17	0.84 ± 0.11	1.12 ± 0.20	1.13 ± 0.19	2.82 ± 0.29 *
*TFAM*	1.30 ± 0.12 *	1.03 ± 0.07	1.20 ± 0.10	1.09 ± 0.09	0.94 ± 0.05
Plasma membrane proteins					
*SLC2A4*	0.91 ± 0.06	0.94 ± 0.09	0.95 ± 0.08	0.84 ± 0.05	0.57 ± 0.10 *
*CAV1*	1.15 ± 0.11	1.15 ± 0.10	1.10 ± 0.12	0.96 ± 0.10	1.35 ± 0.15

*ADIPOQ*—Adiponectin, *LEP*—Leptin, *IL6*—Interleukin 6, *IL1B*—Interleukin 1β, *IL18* – Interleukin 18, *PPARGC1A*—Peroxisome proliferator-activated receptor gamma coactivator 1-alpha, *TFAM*—Mitochondrial transcription factor A, *SLC2A4*—Glucose transporter type 4, *CAV1*—Caveolin 1. Human subcutaneous adipose tissue was incubated without (control) or with Sim or SA. Dexamethasone (Dex)—positive control. Gene expression was normalized to the expression of the housekeeping gene 18S. Relative expression was calculated as 2^−ΔΔCt^. Results are shown as mean ± SEM of *n* = 9 independent experiments. * *p* < 0.05, relative to control.

**Table 6:** Fold change in gene expression in SGBS adipocytes differentiated in the absence or presence simvastatin (Sim) and simvastatin acid (SA).

Differentiation Day						
	**Day 7**			**Day 14**		
Gene symbol	Sim 100 nM	SA 8 nM	SA 30 nM	Sim 100 nM	SA 8 nM	SA 30 nM
Regulation of Adipogenesis						
*PPARG*	0.94 ± 0.12	1.05 ± 0.03	0.89 ± 0.08	1.07 ± 0.09	1.20 ± 0.09	0.93 ± 0.05
Adipokines						
*ADIPOQ*	0.45 ± 0.08 (*p* = 0.058)	0.93 ± 0.03	0.66 ± 0.12	0.66 ± 0.06 (*p* = 0.054)	0.93 ± 0.38	0.41 ± 0.11 (*p* = 0.054)
*LEP*	0.92 ± 0.08	0.89 ± 0.06	0.84 ± 0.05	0.79 ± 0.07	0.80 ± 0.03	1.19 ± 0.06
Regulation of Miochondrial Function						
*PPARGC1A*	0.81 ± 0.12	0.78 ± 0.04 (*p* = 0.06)	0.71 ± 0.06 (*p* = 0.06)	1.03 ± 0.09	1.07 ± 0.26	1.01 ± 0.12
*PDK4*	0.95 ± 0.12	1.02 ± 0.02	1.06 ± 0.22	1.17 ± 0.06	1.04 ± 0.10	0.93 ± 0.03
*TFAM*	0.94 ± 0.05	1.12 ± 0.01 **	0.95 ± 0.06	0.96 ± 0.15	0.97 ± 0.10	0.86 ± 0.16
Plasma Membrane Proteins						
*SLC2A4*	0.62 ± 0.04 *	0.76 ± 0.09	0.76 ± 0.11	0.79 ± 0.16	0.79 ± 0.10	1.06 ± 0.07

*PPARG*—Peroxisome proliferator-activated receptor gamma, *ADIPOQ*—Adiponectin, *LEP*—Leptin, *PPARGC1A*—Peroxisome proliferator-activated receptor gamma coactivator 1-alpha, PDK4—Pyruvate dehydrogenase kinase 4, *TFAM*—Mitochondrial transcription factor A, *SLC2A4*—Glucose transporter type 4. Human SGBS adipocytes were differentiated without (control) or with Sim or SA. Gene expression was normalized to the expression of the housekeeping gene GUSB. Relative expression was calculated as 2^−ΔΔCt^ relative to respective control. Results are shown as mean ± SEM of *n* = 3 independent experiments. * *p* < 0.05, ** *p* < 0.01.

**Table 7:** Biochemical and anthropometric characteristics of the subjects.

Variable	Mean ± SD
*n*	216
Sex (male/female), *n*	73/143
Diabetes status (Healthy/T2D/Prediabetes), *n*	153/47/16
Age (years)	57 ± 14
BMI (kg/m^2^)	30.1 ± 5.9
Waist/hip ratio	0.9 ± 0.1
Systolic blood pressure (mmHg)	135.7 ± 17.5
Diastolic blood pressure (mmHg)	76.5 ± 10.9
Body fat (%)	38.2 ± 9.7
Plasma glucose (mmol/L)	6.4 ± 1.4
HbA1C (mmol/mol)	39.3 ± 9.2
Serum insulin (mU/L)	12.1 ± 8.9
HOMA-IR (mmol × mU/L^2^)	3.4 ± 2.9
Plasma total cholesterol (mmol/L)	4.5 ± 1.8
Plasma HDL cholesterol (mmol/L)	1.8 ± 0.9
Plasma LDL cholesterol (mmol/L)	3.2 ± 0.9
Plasma triglycerides (mmol/L)	1.4 ± 0.7

Data show mean ± SD, HbA1c, glycosylated hemoglobin; HOMA-IR, homeostatic model assessment of insulin resistance index (fasting glucose × fasting insulin/22.5); LDL, low-density lipoprotein; HDL, high-density lipoprotein.

## Data Availability

The data presented in this study are available within the article and Supplementary Material.

## Supplementary Materials

The following are available online at https://www.mdpi.com/article/10.3390/metabo11090574/s1, Figure S1: Adipose tissue pathways positively associated with *HMGCR*, Figure S2: Adipose tissue pathways negatively associated with *HMGCR*. Table S1: List of genes identified in the pathways positively and negatively associated with *HMGCR* in adipose tissue.
