# Supplementary material for: Impaired HMG-CoA Reductase Activity Caused by Genetic Variants or Statin Exposure: Impact on Human Adipose Tissue, β-Cells and Metabolome
Source: Metabolites. 2021 Aug 25;11(9):574. doi: 10.3390/metabo11090574 (PMC8468287; doi:10.3390/metabo11090574)
Supplement: Supplementary file 1 [file metabolites-11-00574-s001.zip › Supplementary Table 1.pdf]

Table S1. List of genes identified in the pathways positively and negatively associated with *HMGCR* in adipose tissue.

	Negatively Associated Genes	Positively Associated Genes
Insulin Signalling Pathways	*PPP1CA, PHKA2, FLOT1, EXOC7, HK1, PRKAB1, G6PC3, PCK2, MTOR, RPS6KB2, RPS6, EIF4EBP1, EIF4E2, BAD, SHC2, HRAS, ARAF, MAP2K2*	*PIK3R1, PIK3CA, PIK3CB, PDPK1, AKT2, GSK3B, PPP1R3C, PPP1R3B, PHKA1, PDE3B, PRKACB, PRKAR2B, PRKCI, SH2B2, CBL, CBLB, CRK, CRKL, RAPGEF1, RHOQ, PRKAA2, PRKAB2, FOXO1, SHC3, SOS1, SOS2, KRAS, BRAF, MAP2K1, ELK1, SOCS3, PTPN1, MAPK8, MAPK10, MAPK9*
Insulin Resistance	*PPP1CA, MTOR, RPS6KB2, NFKBIA, RELA, PRKAB1, SLC27A4, SLC27A5, TRIB3, PCK2, G6PC3, NR1H3, MLXIPL, SLC2A1, CREB3*	*RPS6KA3, PPP1R3C, PPP1R3B, PTPN1, PTPN11, PIK3CA, PIK3CB, PIK3R1, AKT2, IL6, STAT3, SOCS3, GSK3B, TNF, TNFRSF1A, MAPK8, MAPK10, MAPK9, TBC1D4, PRKAA2, PRKAB2, CD36, FOXO1, PRKCE, PDPK1, OGT, GFPT2, GFPT1, CREB3L2*
Adipocytokine Signalling	*TRADD, TRAF2, MTOR, NFKBIA, NFKBIB, RELA, POMC, PRKAB1, PCK2, G6PC3, RXRA, SLC2A1*	*TNF, TNFRSF1A, MAPK8, MAPK10, MAPK9, SOCS3, AKT2, CD36, ACSL3, STAT3, PRKAA2, PRKAB2, PTPN11, ADIPOQ, ADIPOR2*
