# Supplementary figures and images for: Impaired HMG-CoA Reductase Activity Caused by Genetic Variants or Statin Exposure: Impact on Human Adipose Tissue, β-Cells and Metabolome
Source: Metabolites. 2021 Aug 25;11(9):574. doi: 10.3390/metabo11090574 (PMC8468287; doi:10.3390/metabo11090574)

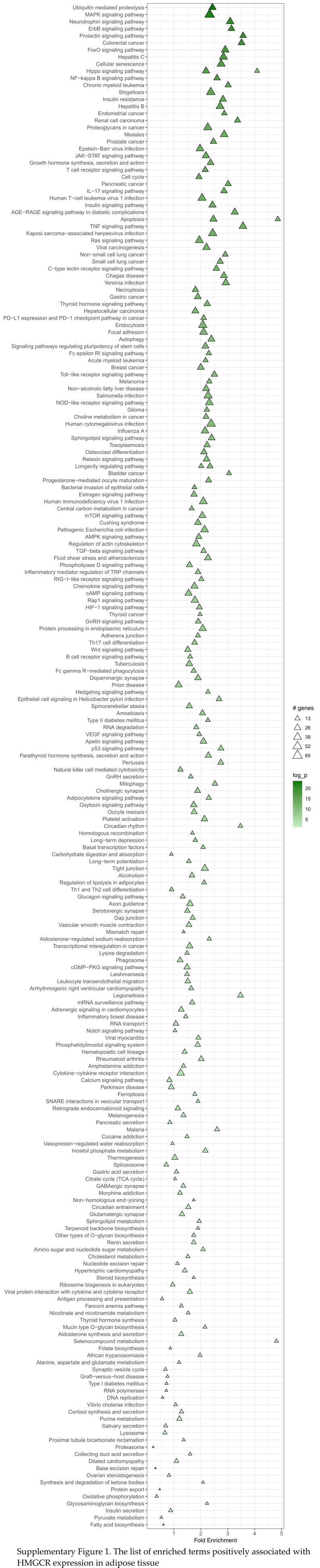

Supplement: Supplementary file 1 [file metabolites-11-00574-s001.zip › Suppl.Fig1 revision.png]

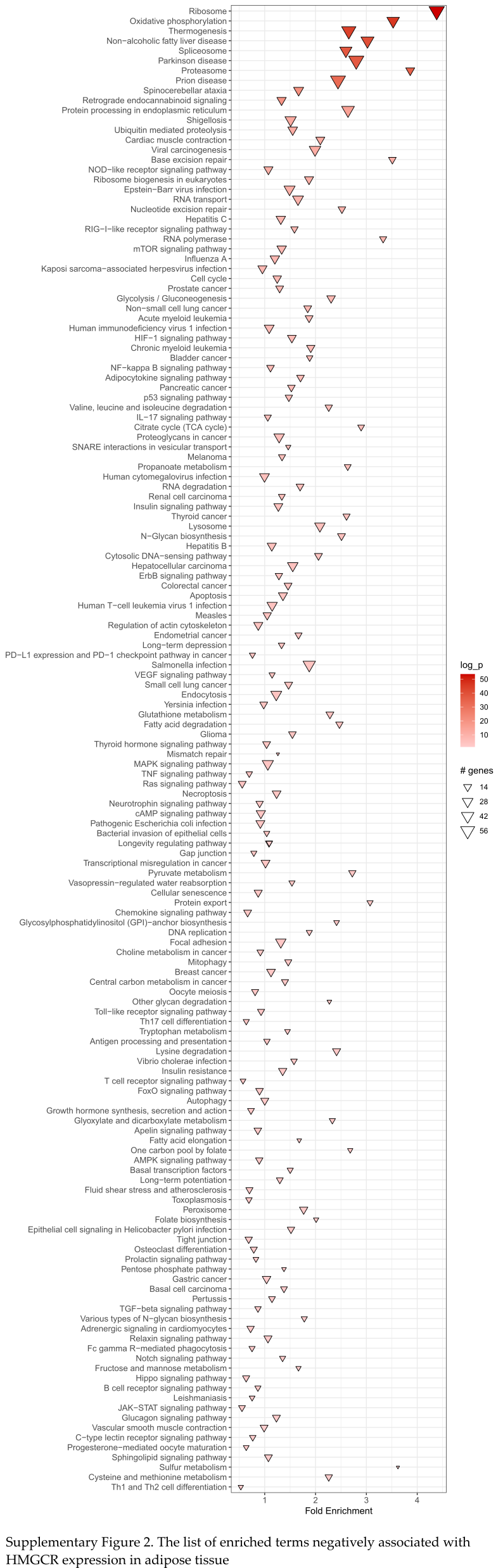

Supplement: Supplementary file 1 [file metabolites-11-00574-s001.zip › Suppl.Fig2 revision.png]
